# Extensive intramuscular manifestation of sarcoidosis with initially missed diagnosis and delayed therapy: a case report

**DOI:** 10.1186/s13256-017-1403-3

**Published:** 2017-08-24

**Authors:** Niklaus Meyer, Reto Sutter, Udo Schirp, Andreas Gutzeit

**Affiliations:** 1 Institute of Surgery, Kantonsspital Winterthur, Brauerstrasse 15, 8401 Winterthur, Switzerland; 2 Institute of Radiology, University Hospital Balgrist, Forchstrasse 340, 8008 Zürich, Switzerland; 3 Institute of Radiology and Nuclear Medicine, Hirslanden Klinik St. Anna, St. Anna-Strasse 32, 6006 Lucerne, Switzerland; 4 Department of Radiology, Paracelsus Medical University, Strubergasse 21, 5020 Salzburg, Austria; 5 Department of Chemistry and Applied Biosciences, ETH Zurich, Vladimir Prelog Weg 1 -5/10, 8093 Zurich, Switzerland

**Keywords:** Sarcoidosis, Myositis, Muscle sarcoidosis

## Abstract

**Background:**

Sarcoidosis is a multisystemic granulomatous disorder, which in nearly all cases involves the lungs and other organs. Isolated forms of sarcoidosis within the muscles, but without lung involvement, are extremely rare and can lead to delayed or even false diagnosis.

**Case presentation:**

A 52-year-old white, Swiss man presented with painful arm cramps and a history of symptoms over the previous 3 years. In the initial clinical investigation, our patient also showed edema in both legs without any other complaints. After performing an magnetic resonance imaging scan of his extremities and a positron emission tomography/computed tomography scan, diffuse myositis was described. The subsequent muscle biopsy provided the surprising diagnosis of muscle sarcoidosis, without involvement of the lungs or any other organ. After starting therapy with glucocorticoids, his symptoms improved immediately.

**Conclusions:**

Sarcoidosis is a common disorder, which in most cases affects the lungs. In this case report an isolated sarcoidosis is described without lung involvement, but with involvement of the muscles of the extremities and the trunk. Reported cases of sarcoidosis only involving skeletal muscle and without lung involvement are extremely rare. Radiologists should consider this presentation of sarcoidosis to avoid delayed diagnosis and therapy.

## Background

Sarcoidosis is a multisystem granulomatous disorder, which was first described by Hutchinson in London 1877 [1]. Sarcoidosis may involve any organ and clinically presents with diverse manifestations involving accumulation of T lymphocytes, mononuclear phagocytic cells and non-caseating granulomas in a number of organs [2]. In more than 90% of cases, the lungs are involved. Extrapulmonary sarcoidosis, in combination with lung involvement, is found in 30% of patients [3]. The most common sites of extrapulmonary disease include the skin, eyes, exocrine glands, heart, kidneys and central nervous system [4, 5]. Extrapulmonary sarcoidosis without lung involvement is very rare.

Secondary musculoskeletal manifestations of sarcoidosis occur in 20% of patients with sarcoidosis and include joint involvement, bone lesions, and muscular disease [6–11]. Historically, there have only been two reported cases which describe the isolated sarcoidosis manifestation of muscles, without involvement of the lungs. The first case was in 1961 and the second in 1994 [12, 13]. In the current case report, we present a clinical situation of isolated muscle sarcoidosis involving the trunk and extremities, but without lung involvement. After initial misinterpretation the sarcoidosis was diagnosed with positron emission tomography/computed tomography (PET/CT) and magnetic resonance imaging (MRI).

## Case presentation

A white Swiss man presented with painful arm cramps and a history of symptoms over a period of 3 years. In the initial clinical investigation, the 52-year-old carpenter also showed edema in both legs, without any other complaints. In his personal history, the patient had a history of a seronegative non-tropical sprue as well as a history of several orthopedic procedures including total hip arthroplasty.

A physical examination revealed full and painless range of motion of all joints of the upper and lower extremities with normal muscle strength. His serum human leukocyte antigen-B27 (HLA-B27) result was positive and his urine acid was mildly elevated (467 U/l). Other screening laboratory examinations, such as antinuclear antibodies (ANA), anti-dsDNA antibodies, rheumatoid factor (RF), anti-citrullinated peptide antibodies (anti-CCP), serology for borreliosis and thyroid-stimulating hormone (TSH), were negative.

**Fig. 1:**
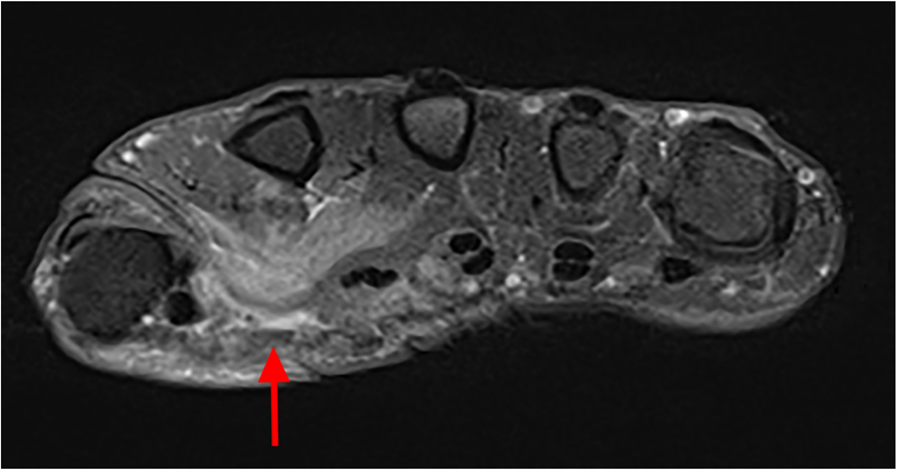
A magnetic resonance imaging examination of his right hand at initial presentation. T1-weighted fat-saturated axial magnetic resonance image shows diffuse enhancement of the muscles and the palmar aponeurosis (*arrow*). There are no pathological features in his bones or joints

Radiographs of his hands and feet and a chest radiograph remained unremarkable. An initial MRI scan of both hands showed a diffuse contrast enhancement of the palmar aponeurosis and muscles (Fig. 1). His joints and bones did not show any pathological changes.

An empiric therapy was started with prednisolone 20 mg for 10 days and 10 mg daily for an additional 6 weeks. Our patient showed complete resolution of pain complaints after 6 weeks and therapy was discontinued. A follow-up consultation was planned after 6 months and cancelled by the asymptomatic patient.

One year after the first presentation, our patient presented again to our hospital with reduced general health and a diffused weakness of his extremities. In addition to recurrent pain in his arms and hands, our patient also showed a new symptom – weakness and pain in his upper and lower legs.

**Fig. 2:**
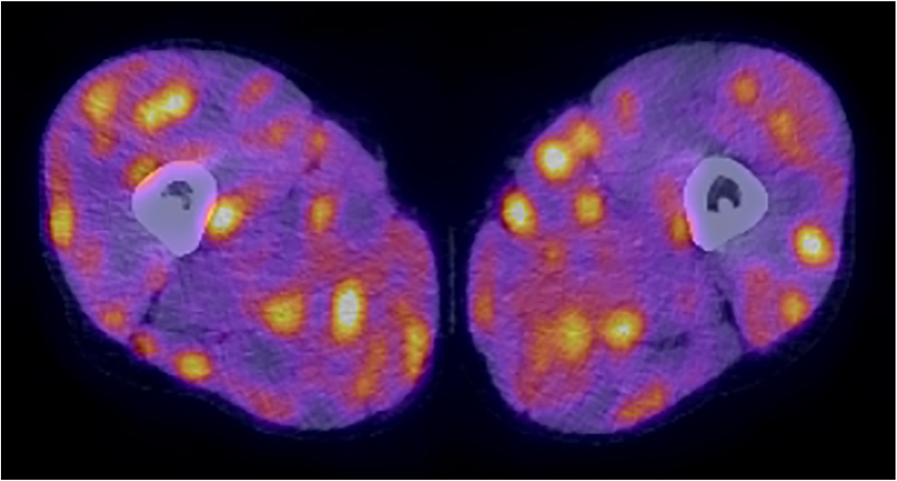
A positron emission tomography/computed tomography examination of the upper thighs 1 year after initial presentation. An axial positron emission tomography/computed tomography image demarks diffuse bilateral distribution of spots with increased fludeoxyglucose uptake of all muscles of the upper thighs

**Fig. 3:**
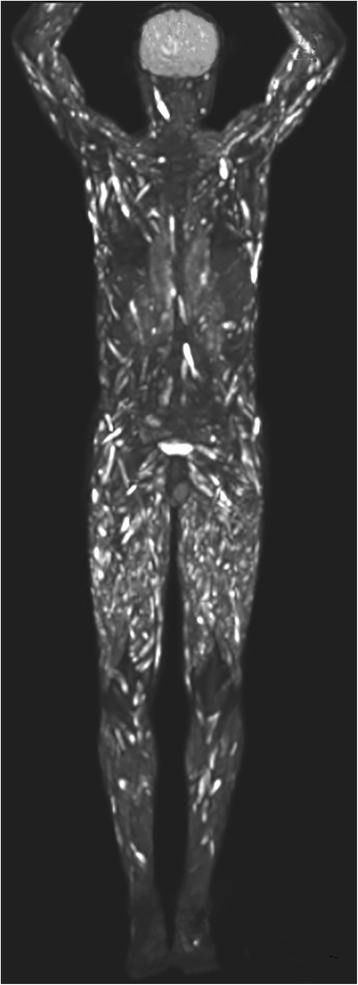
Coronal reconstruction of a positron emission tomography/computed tomography examination shows increased fludeoxyglucose uptake with diffuse involvement of the muscles of the extremities and the trunk

Due to these generalized symptoms and lack of diagnostic evidence, a whole-body PET-CT scan was performed. A diffused uptake of fludeoxyglucose (FDG) was found in all skeletal muscles of his legs and arms without involvement of his lungs or lymph nodes (Figs. 2 and 3). Furthermore, various other muscles of his chest and body showed unspecific FDG uptake (Fig. 3). Unspecific myositis was suspected at this point. In order to confirm the diagnosis, a transcutaneous muscle biopsy was performed in the quadriceps muscle of his right leg. The biopsy of the affected muscles showed granulomatous myositis.

Based on the diagnosis of sarcoidosis of the skeletal muscle, a therapy with prednisolone 50 mg/day was started again. This therapy reduced the muscle complaints immediately. In order to preserve the good clinical condition of our patient, a weekly, supporting therapy with methotrexate (20 mg) and folic acid (5 mg) was established and administration of prednisolone was tapered. After 1 year, our patient was symptom-free and was able to work without any restrictions. No further imaging was performed due to the positive clinical outcome.

## Discussion

Sarcoidosis is a systemic granulomatous disease and the diagnosis is based on a clinico-radiological correlation and histological evidence of non-caseating granulomas. In most cases, lungs are involved primarily and other organs may be affected secondarily. In this case report, we describe an uncommon form of isolated sarcoidosis, involving skeletal muscles of the extremities exclusively without involvement of lungs or other visceral organs or bones.

Lungs and thoracic lymph nodes in patients with sarcoidosis are involved in 95–100% of all cases [14]. In such a combination, the diagnosis of sarcoidosis is relatively simple. In most cases, the diagnosis is confirmed after biopsy of the lymph nodes of the mediastinum or after performing a bronchoalveolar lavage.

In rare cases, sarcoidosis has been shown to involve organs, but sparing the lungs [15]. In such cases, an incorrect interpretation of the findings and delayed treatment can be the result. There have only been two cases previously described, reporting isolated sarcoidosis of the muscle without involvement of the lungs [12, 13]. Due to the fact that the lung is generally affected in sarcoidosis, the radiological staging system is still based on chest radiographs even though for the detection of parenchymal involvement high-resolution computer tomography (HRCT) is required. In the case of sarcoidosis outside of the lungs, cross-sectional examinations such as PET/CT and MRI are useful. It is therefore important that radiologists are aware of atypical disease patterns, in order to interpret these accurately and effectively.

In most cases, musculoskeletal involvement of sarcoidosis is associated with lung involvement. Musculoskeletal sarcoidosis mostly involves bones. The typical pattern of such a disease involves lytic lesions of the bones [15]. These lytic lesions are quite typical and specific diagnoses are possible with radiographs. MRI of nodular musculoskeletal sarcoidosis typically revealed a well-demarcated nodule with a heterogeneous signal of the bone [12, 15, 16].

In this special situation, we describe a case of sarcoidosis involving the muscles of the extremities and trunk without any involvement of the lungs or bones. Because of this unusual situation, it was more challenging to make an accurate diagnosis and to determine an appropriate treatment.

As described in Fig. 1, there was only contrast enhancement with the pattern of myositis. Due to the false assumption of atypical myositis, a PET/CT scan was performed during the course of the disease. The PET/CT scan confirmed extensive myositis without involvement of the bones or other organs. Only the muscle biopsy made the diagnosis evident.

We hope that this case report will help radiologists to better interpret these atypical courses so that the patient can be diagnosed and treated without any delay. Such imaging should suggest a biopsy in similar situations in order to secure a accurate diagnosis.

## Conclusion

Isolated sarcoidosis manifestation within the muscles without involvement of the lungs is extremely rare. This should be considered when myositis presents in an atypical form. In order to confirm the diagnosis a muscle biopsy should be performed.

## Abbreviations

ANA: Antinuclear antibodies
Anti-CCP: Anti-citrullinated peptide antibodies
FDG: Fludeoxyglucose
HRCT: High-resolution computer tomography
MRI: Magnetic resonance imaging
PET/CT: Positron emission tomography/computed tomography
RF: Rheumatoid factor
Serum HLA-B27: Human leukocyte antigen-B27
TSH: Thyroid-stimulating hormone
